# Comparison Study of Manometric Respirometric Test  and Common Chemical Methods in the Determination of ${\text{BOD}}_{\text{bold7}}$ in a Pulp and Paper Mill's Wastewaters

**DOI:** 10.1155/JAMMC/2006/90384

**Published:** 2006

**Authors:** Katri Roppola, Toivo Kuokkanen, Hannu Nurmesniemi, Jaakko Rämö, Risto Pöykiö, Hanna Prokkola

**Affiliations:** 1 Department of Chemistry University of OuluP.O. Box 3000 Oulu 90014 Finland; 2 Stora Enso Oyj Veitsiluoto Mills Kemi 94800 Finland; 3 Water Resources and Environmental Engineering Laboratory University of Oulu P.O. Box 4300 Oulu 90014 Finland; 4 The Town Planning Committee The Environmental Research Division City of Kemi Valtakatu 26 Kemi 94100 Finland

## Abstract

The biological oxygen demand (BOD) test is widely used in many wastewater treatment plants. The conventional BOD tests are usually time-consuming and the results are often out of date for process control purposes. The aim of this research was to compare the manometric respirometric test with common chemical methods in the determination of BOD of wastewater from a pulp and  paper mills as well as to evaluate the BOD _7_ values of both wastewaters from the short-term respirometric measurements. The results showed that there were differences in the  BOD_7_ values of paper mill samples measured by conventional and  respirometric methods. The main cause was found to be the dilution solution used in the conventional BOD tests. Using the same mineral solution in the respirometric measurements diminished the difference remarkably.  Evaluation of the  BOD_7_ value after two or three days incubation  was proved to work very well and the estimated results were close to measured values (deviations  1%–12%).


Hindawi Publishing Corporation
Journal of Automated Methods and Management in Chemistry
Volume 2006, Article ID 90384, Pages 1–5
DOI 10.1155/JAMMC/2006/90384

### Comparison Study of Manometric Respirometric Test and Common Chemical Methods in the Determination of $BOD_7$ in a Pulp and Paper Mill's Wastewaters

**Katri Roppola,[1] Toivo Kuokkanen,[1] Hannu Nurmesniemi,[2] Jaakko Rämö,[3] Risto Pöykiö,[4] and Hanna Prokkola[1]**

[1] *Department of Chemistry, University of Oulu, P.O. Box 3000, 90014 Oulu, Finland*
[2] *Stora Enso Oyj Veitsiluoto Mills, 94800 Kemi, Finland*
[3] *Water Resources and Environmental Engineering Laboratory, University of Oulu, P.O. Box 4300, 90014 Oulu, Finland*
[4] *The Town Planning Committee, The Environmental Research Division, City of Kemi, Valtakatu 26, 94100 Kemi, Finland*

Received 23 November 2005; Revised 29 June 2006; Accepted 6 September 2006

The biological oxygen demand (BOD) test is widely used in many wastewater treatment plants. The conventional BOD tests are usually time-consuming and the results are often out of date for process control purposes. The aim of this research was to compare the manometric respirometric test with common chemical methods in the determination of BOD of wastewater from a pulp and paper mills as well as to evaluate the $BOD_7$ values of both wastewaters from the short-term respirometric measurements. The results showed that there were differences in the $BOD_7$ values of paper mill samples measured by conventional and respirometric methods. The main cause was found to be the dilution solution used in the conventional BOD tests. Using the same mineral solution in the respirometric measurements diminished the difference remarkably. Evaluation of the $BOD_7$ value after two or three days incubation was proved to work very well and the estimated results were close to measured values (deviations 1%–12%).

Copyright © 2006 Katri Roppola et al. This is an open access article distributed under the Creative Commons Attribution License, which permits unrestricted use, distribution, and reproduction in any medium, provided the original work is properly cited.

### 1. INTRODUCTION

The biological oxygen demand (BOD) is a measure for the quantity of oxygen required for the biodegradation of organic matter in water. The BOD tests have been used for over a century to determine the amount of biodegradable organic matter in wastewater. Although the BOD is an essential sum parameter in water management, conventional BOD measuring tests are very slow, typically five or seven day long. The five-day lasting BOD test was developed about a hundred years ago in England on the basis of the maximum time required for any British river to flow from its source to the sea [1]. The long-lasting measuring time causes problems controlling a wastewater treatment plant because the wastewater in the treatment plant has already changed during the test. Accordingly, the results are ordinarily out of date for process control or monitoring purposes. Time-consuming test like iodometric method [2] or electrochemical probe method [3] is also distorted by dilution and sample preparation.

The $BOD_5$ or $BOD_7$ values of wastewaters have been tried to estimate with several short-term tests, for example, different kinds of sensors [4, 5]. Improved techniques enable us to measure BOD using faster and more precise tests like automatic manometric respirometric test that we have used in this research. In this work we show that the manometric respirometric test has many advantages compared with the classical BOD methods including reduced sample preparation time, use of nondiluted samples, easy and continuous reading of the measuring data, and faster measuring time. This respirometric test is based on automatic pressure measurement in a closed bottle under constant temperature. Micro-organisms consume oxygen by degrading organic matter and the formed $CO_2$ gas is chemically bound by the sodium hydroxide pellets. The overall result is a pressure decrease in the bottle. The instrument calculates automatically the BOD value using the ideal gas law modified for conditions in a closed space. The BOD value can be read continuously during the test [6].

The aim of the present study was to compare the manometric respirometric test with conventional chemical methods in the determination of BOD of wastewater and to estimate the applicability of the respirometric test in the determination of BOD of wastewater from pulp and paper mills. In addition, the other objective was to estimate the seventh-day BOD value after a short-term measuring period (1–3 days) by respirometric test. Conventional chemical methods give only one BOD result after the seven day incubation. Predictability of the $BOD_7$ value will reduce the measurement time improving, for example, the chemical adjustment of the wastewater treatment process. The wastewater samples were collected from Stora Enso Oyj Veitsiluoto Mills at Kemi. The samples were taken from both the sewer of the chemical pulp mill and the sewer of the paper mill. Just recently we have studied the biodegradation of different oils in groundwater and conditions described in OECD 301F standard using the manometric respirometric method [7–9]. These results showed that the respirometric method is very suitable for biodegradation studies of oils in water, although the test has been developed for BOD measurements of municipal wastewaters. On the other hand, this study is a new part of the major project focusing on the effects of the pulp and paper mills' effluents on the aquatic environment in Northern Finland [10].

### 2. EXPERIMENTAL

### 2.1. Theory of the measurements

A manometric respirometric test was carried out with the OxiTop Control system [6]. The test is based on very accurate automatic pressure measurement in a closed bottle under constant temperature (20 ± 0.2°C). A certain amount of oxygen is consumed during the biodegradation process of the organic matter. At the same time the formed $CO_2$ gas is removed from the gas space by means of an absorber (NaOH) so that the resulting pressure decline is a measure of the biological oxygen demand. The instrument calculates BOD in mg/L using the ideal gas law modified for conditions in a closed space:

$$\text{BOD}\,[\text{mg L}^{-1}] = \text{M}(\text{O}_2)/RT_m \cdot \left[(V_{\text{tot}} - V_l)/V_l + aT_m/T_0\right] \cdot \Delta p(\text{O}_2), \quad (1)$$

where $\text{M}(\text{O}_2)$ is molar mass of oxygen (32.0 g $\text{mol}^{-1}$), $R$ is the gas constant (83.144 L hPa $\text{mol}^{-1}\,\text{K}^{-1}$), $T_m$ is the measuring temperature (293.15 K), $T_0$ is 273.15 K, $V_{\text{tot}}$ is the bottle volume (mL), $V_l$ is the liquid phase volume (mL), $a$ is the Bunsen absorption coefficient (0.03103), and $\Delta p(\text{O}_2)$ is the difference in partial oxygen pressure (hPa).

Before the measurement it is essential to estimate the measuring range of the sample to be analyzed. That determines the amount of water used. For example, when a measurement scale is 0–80 mg $\text{L}^{-1}$, 365 mL of sample is needed [6]. If a nitrification inhibitor and/or an extra microbe source are used, they must be added before filling the measuring flask in order to keep the overall sample volume constant. Effect of sample dilution by mineral nutrient solution on result was investigated in the present study.

### 2.2. Wastewater samples examined in the study

Wastewater samples used in this research were collected from Stora Enso Oyj Veitsiluoto Mills at City of Kemi, Northern Finland. The Veitsiluoto mills produce annually approximately 365 000 tonnes of bleached softwood and hardwood pulps, 555 000 tonnes of uncoated fine paper, 370 000 tonnes of paper sheets, 420 000 tonnes of coated paper, and 290 000 cubic metres of sawn wood [10].

The wastewater samples were collected during seven separate days in 2005. The samples were collected from a sewer of the chemical pulp mill after biological treatment and sewer of the paper mill after chemical polyaluminium treatment. The quality of the studied water samples was widely diverse. For example, according to long-term follow-up studies in Stora Enso Oyj, wastewater from the pulp mill has larger solid matter and metal contents than the wastewater from the paper mill. The dissimilarity between these two wastewaters was the one selection criterion for the samples of this study.

Nitrification is a two-step respiratory process in which bacteria oxidise ammonium to nitrite and nitrate. The wastewater from the forest industry contains very little total nitrogen and very little free ammonia that can be oxidised to nitrite/nitrate. It is estimated that the maximum contribution of ammonia oxidation to the total BOD observed is less than 5% [11].

### 2.3. Measurement of BOD using manometric respirometric test

A measurement region of 0–40 mg/L was chosen for the chemical pulp mill samples and a measurement region of 0–80 mg/L for the diluted and undiluted paper mill samples. All measurements by the chemical reference methods used in this study were carried out with diluted samples using dilution factors 5 and 20 for the pulp mill and the paper mill samples, respectively. Therefore, the effect of dilution on the BOD value was wanted to test also with the manometric respirometric test. The samples were diluted with nutrient solution applying to SFS 1889-1 standard [12] using the dilution factor 2. The nutrient solution was prepared using $KH_2PO_4$, $K_2HPO_4$, $Na_2HPO_4 \cdot 2H_2O$, $NH_4Cl$, $CaCl_2$, $MgSO_4 \cdot 7H_2O$, and $FeCl_3 \cdot 6H_2O$. According to long-term mill experience, the role of nitrification in oxygen consumption is minor and to follow the common mill practice, nitrification inhibitor *n*-allylthiourea (ATU) was not used in all measurements. To confirm this low nitrification rate, however, a few experiments were carried out using also the nitrification inhibitor ATU. The bottles were sealed with a rubber sleeve containing a $CO_2$ absorber. The measuring heads were screwed onto the bottles and the samples were stabilised in the incubation cabinet (20.0 ± 0.2°C) for six hours before the measurement was started.

### 2.4. Measurement of BOD using conventional chemical methods

The used chemical reference method was *determination of biochemical oxygen demand after n days* ($\text{BOD}_n$). Part 1; *dilution and seeding method with allylthiourea addition* [12]. The

Table 1: The $BOD_7$ values determined by respirometry, iodometric method, and oxygen sensor for wastewater samples originated from the Stora Enso Oyj Veitsiluoto Mills.

| Sample | Respirometry BOD [mg $L^{-1}$] | Iodometric method BOD[mg $L^{-1}$] | Oxygen sensor BOD[mg $L^{-1}$] |
|---|---|---|---|
| Chem. pulp (I ) | 22 | 22 | 16 |
| Paper(I) | 50 | 81 | 73 |
| Chem. pulp (II) | 33 | 19 | 15 |
| Paper mill (II) | 30 | 79 | 58 |
| Chem. pulp (III) | 38 | 20 | 19 |
| Paper (III) | 58 | 110 | 92 |
| Chem. pulp (IV) | 30 | 17 | 19 |
| Paper (IV) | 67 | 100 | 95 |
| Chem. pulp (V) | 37 | 19 | 23 |
| Paper (V) | 70 | 120 | 130 |
| Chem. pulp (VI) | 19 | 13 | 11 |
| Paper (VI) | 63 | 130 | 110 |

(I), (II), (III), and so forth = samples taken in different times.

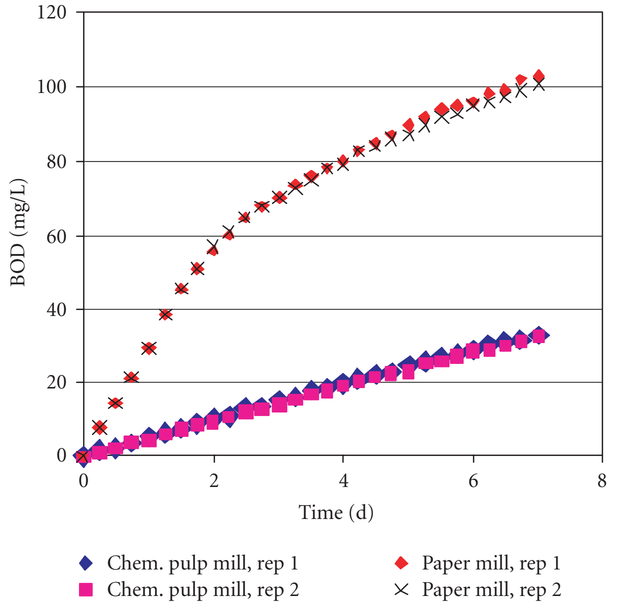

Figure 1: The respirometric $BOD_7$ diagrams of the wastewater samples originated from the Stora Enso Oyj Veitsiluoto Mills.

residual oxygen after seven days incubation was determined according to standards *determination of dissolved oxygen, iodometric method* [2], and *electrochemical probe method* [3].

To ensure reproducibility, analyses with an oxygen sensor were carried out in two separate laboratories. The analyses with an oxygen sensor (WTW, Stirrox G, WP3-ST) were carried out in the laboratory of the mills and the other measurements were performed in the University of Oulu. In order to eliminate the measuring errors some test measurements with an oxygen sensor (WTW, Cellox, 325) were carried out in the University of Oulu. These results proved that measurements were reliable. The pH values of the wastewater samples were measured with a Consort P600 pH meter.

### 2.5. Evaluation of the $BOD_7$ values

The BOD values were registered in every second hour and the developed graphs are in general mathematically regular in shape. So, the seventh-day BOD value can be evaluated already in the early stage of the measurement (e.g., after 1–3 days). Computer programs used in this study were Excel, Sigma Plot, and TBL-Curve.

### 3. RESULTS AND DISCUSSION

### 3.1. Comparison of different methods and the effect of dilution on the BOD value

The BOD values of the samples, as determined by the different methods, are presented in Table 1.

For the paper mill samples, the respirometric method gave lower BOD results as compared with the chemical methods. One possible explanation is lack of nutrients in this chemically treated wastewater. According to this, the nutrients were provided only by the dilution solution. The effect of dilution solution on the BOD values measured by respirometry was thus investigated. Dilution factor 2 was used for the respirometric measurements. Table 2 reveals that BOD values for all diluted samples are very similar and independent on dilution factor and method. Adequate amount of nutrients is provided using dilution factor 2 and increasing of that seems to have no increasing effect on results. BOD values measured from undiluted samples differ completely from those measured from the diluted samples. These values, however, represent the real mill situation. This must be notified when interpreting the results and can be addressed as a clear advantage of manometric respirometric method.

The dilution of the pulp mill samples has no significant effect on BOD values probably because these samples naturally contain a sufficient amount of nutrients for the biodegradation process. This can be observed in Table 2 which reveals no difference between dilution factors 1 and 2 when measuring by respirometry. The values measured with the respirometric method were larger in every case compared to those measured by the chemical methods. It is possible that volatile compounds, like methanol, mercaptans, and small organic acids, which are typical in activated sludge processes, absorb into sodium hydroxide. This may cause an additional decrease in measured pressure and thus overestimated result. This potential pitfall should be notified when interpreting the results in process control purposes. The phenomenon will be focused on in further research by the authors in a part of wide project in collaboration with the forest industry.

The precision of the respirometric test was determined by measuring the BOD values of the wastewater samples twice. Figure 1 shows typical results of duplication tests for the

Table 2: The effect of dilution on the $BOD_7$ values in respirometric measurements.

| Sample | Dilution factor in respirometric measurements | Respriometry $BOD_7$ [mg $L^{-1}$] | Iodometric method* $BOD_7$ [mg $L^{-1}$] | Oxygen sensor* $BOD_7$ [mg $L^{-1}$] |
|---|---|---|---|---|
| Paper (III) | 1 | 58 | — | — |
| Paper (III) | 2 | 110 | 110 | 92 |
| Chem. pulp (IV) | 1 | 30 | — | — |
| Chem. pulp (IV) | 2 | 28 | 17 | 19 |
| Paper (IV) | 1 | 67 | — | — |
| Paper (IV) | 2 | 100 | 100 | 95 |
| Chem. pulp (V) | 1 | 37 | — | — |
| Chem. pulp (V) | 2 | 39 | 19 | 26 |
| Paper (V) | 1 | 70 | — | — |
| Paper (V) | 2 | 120 | 120 | 130 |
| Paper (VI) | 1 | 63 | — | — |
| Paper (VI) | 2 | 100 | 130 | 110 |

*Measurements with iodometric method and oxygen sensor were carried out using dilution factors 5 and 20 for pulp mill and paper mill samples, respectively.

Table 3: Evaluation results of the $BOD_7$ values.

| Sample | Evaluation of $BOD_7$ after 2 days incubation | Evaluation of $BOD_7$ after 3 days incubation | Evaluation of $BOD_7$ after 4 days incubation | Equation number | $BOD_7$ value measured by Respirometry |
|---|---|---|---|---|---|
| Chem. pulp (II) | 32.7 mg $L^{-1}$ | 33.5 mg $L^{-1}$ | 33.1 mg $L^{-1}$ | 1 | 32.5 mg $L^{-1}$ |
| Chem. pulp (III) | 37.4 mg $L^{-1}$ | 37.6 mg $L^{-1}$ | 38.8 mg $L^{-1}$ | 1 | 37.9 mg $L^{-1}$ |
| Chem. pulp (V) | 32.1 mg $L^{-1}$ | 34.8 mg $L^{-1}$ | 35.9 mg $L^{-1}$ | 1 | 36.5 mg $L^{-1}$ |
| Chem. pulp (VI) | 20.4 mg $L^{-1}$ | 20.2 mg $L^{-1}$ | 19.8 mg $L^{-1}$ | 1 | 19.2 mg $L^{-1}$ |
| Paper (IV) | 114 mg $L^{-1}$ | 94.7 mg $L^{-1}$ | 92.2 mg $L^{-1}$ | 2 | 102 mg $L^{-1}$ |
| Paper (V) | 129 mg $L^{-1}$ | 120 mg $L^{-1}$ | 119 mg $L^{-1}$ | 2 | 121 mg $L^{-1}$ |
| Paper (VI) | 96 mg $L^{-1}$ | 97 mg $L^{-1}$ | 96 mg $L^{-1}$ | 2 | 103 mg $L^{-1}$ |

*Equation number 1*: $y = ax + b$.
*Equation number 2*: $y = [(a + cx)/(1 + bx)]^2$.

paper mill and the chemical pulp mill samples. The precision of the manometric respirometric test was very good. Standard deviations for chemical pulp mill and paper mill samples were 0.8% and 3.7%, respectively.

A few experiments were carried out by respirometric test using the nitrification inhibitor. The measured values for the pulp mill sample were 33.4 mg/L with ATU and 36.5 mg/L without ATU. Corresponding values for the paper mill samples were 115.6 mg/L and 121.2 mg/L, respectively. The values were thus within 10% and confirmed the mill experience that significant nitrification process did not take place in pulp and paper mill's wastewaters [11].

### 3.2. Evaluation of the $BOD_7$ values

The evaluation of the $BOD_7$ values was calculated with the Sigma Plot or TBL-curve programs (paper mill samples) and Excel program (chemical pulp mill samples). The BOD graphs formed in the respirometric measurements of wastewater from the chemical pulp mill were linear in shape, whereas the diagrams of wastewater from the paper mill were clearly nonlinear (Figure 1). The evaluation results are represented in Table 3.

The results reveal that the estimated $BOD_7$ values for the chemical pulp mill samples were very accurate. Also estimation of the $BOD_7$ values of paper mill samples worked well. As early as after two day incubation, the estimated $BOD_7$ values for both samples were reliable. The difference between the evaluated $BOD_7$ value (after two day incubation) and by respriometric measured value was only from 1 to 12 percent in both cases. As can be seen from Figure 1, the shape of the BOD diagram is individual for different wastewaters. Therefore, fitting and selecting the equation used in evaluation of BOD value is case-specific for the studied wastewaters.

### 4. CONCLUSION

The manometric respirometric test is an accurate, handy, and practical method for the determination of BOD of wastewater from pulp and paper mills. The results showed that there was some deviation between the $BOD_7$ values measured by the manometric respirometric method and conventional chemical methods like iodometric titration or an oxygen sensor. The results also showed that dilution of wastewater samples could have very large effect on $BOD_7$ value. A larger effect was noticed with the paper mill samples, which naturally have a low nutrient content. When the paper mill samples were diluted with mineral solution, the respirometric $BOD_7$ results came close to the results measured with iodometric titration or an oxygen sensor.

Evaluation of the $BOD_7$ value from the short-term measuring results worked very well. The predictability of the $BOD_7$ value by the manometric respirometric method will reduce the measurement time improving, for example, the chemical adjustment of the wastewater treatment process. It must be noted that the predictions should not be used for monitoring against regulatory BOD limits. The differences between the evaluated $BOD_7$ value and measured value after seven days incubation were only from 1 to 12 percent in all cases. Unlike the paper mill samples, the chemical pulp mill samples gave linear BOD graphs. Because of the dissimilarity between the formed curves, the evaluation has been done using the different equations.

### ACKNOWLEDGMENTS

The authors would like to thank Ph.D. holder Pekka Vähäoja for stimulating discussions and Suomen Ympäristöpalvelu Oy (M.Sc. Ilkka Välimäki) for the opportunity to use their WTW OxiTop instrument.

### REFERENCES

[1] P. J. LeBlank, "Review of rapid BOD test methods," *Journal of the Water Pollution Control Federation*, vol. 46, pp. 2202–2208, 1974.

[2] SFS-EN 25813, Water quality. Determination of dissolved oxygen. Iodometric method.

[3] SFS-EN 25814, Water quality. Determination of dissolved oxygen. Electrochemical probe method.

[4] J. Liu and B. Mattiasson, "Microbial BOD sensors for wastewater analysis," *Water Research*, vol. 36, no. 15, pp. 3786–3802, 2002.

[5] S. Rastogi, P. Rathee, T. K. Saxena, N. K. Mehra, and R. Kumar, "BOD analysis of industrial effluents: 5 days to 5 min," *Current Applied Physics*, vol. 3, no. 2-3, pp. 191–194, 2003.

[6] WTW Weilheim, Germany; OxiTop Instruction Manual.

[7] T. Kuokkanen, P. Vähäoja, I. Välimäki, and R. Lauhanen, "Suitability of the respirometric BOD Oxitop method for determining the biodegradability of oils in ground water using forestry hydraulic oils as model compounds," *International Journal of Environmental Analytical Chemistry*, vol. 84, no. 9, pp. 677–689, 2004.

[8] P. Vähäoja, T. Kuokkanen, I. Välimäki, S. Vuoti, and P. Perämäki, "Biodegradabilities of some chain oils in groundwater as determined by the respirometric BOD OxiTop method," *Analytical and Bioanalytical Chemistry*, vol. 381, no. 2, pp. 445–450, 2005.

[9] P. Vähäoja, P. Piltonen, A. Hyvönen, J. Niinimäki, J. Jalonen, and T. Kuokkanen, "Biodegradability studies of certain wood preservatives in groundwater as determined by the respirometric BOD OxiTop method," *Water, Air, and Soil Pollution*, vol. 165, no. 1–4, pp. 313–324, 2005.

[10] R. Pöykiö, E. Taskila, P. Perämäki, et al., "Sediment, perch (Perca fluviatilis L.) and bottom fauna as indicators of effluent discharged from the pulp and paper mill complex at Kemi, Northern Finland," *Water, Air, and Soil Pollution*, vol. 158, no. 1, pp. 325–343, 2004.

[11] Oy Metsä-Botnia ja Stora Enso Oyj, Ympäristölupahakemukseen liittyvä vesistöselvitys.

[12] SFS-EN 1899-1, Water quality. Determination of biochemical oxygen demand after n days ($BOD_n$). Part 1: dilution and seeding method with allylthiourea addition.

